# Sequence investigation of 34 forensic autosomal STRs with massively parallel sequencing

**DOI:** 10.1038/s41598-018-24495-9

**Published:** 2018-05-01

**Authors:** Suhua Zhang, Yong Niu, Yingnan Bian, Rixia Dong, Xiling Liu, Yun Bao, Chao Jin, Hancheng Zheng, Chengtao Li

**Affiliations:** 1 Shanghai Key Laboratory of Forensic Medicine, Shanghai Forensic Service Platform, Academy of Forensic Sciences, Ministry of Justice, Shanghai, 200063 P.R. China; 2 Criminal Investigation Department, Ministry of Public Security, Beijing, 100741 P.R. China; 3 The Affiliated Guangji Hospital of Soochow University, Suzhou, 215008 P.R. China; 4 Shanghai OE Biotechnology Co, Ltd, Shanghai, 201114 P.R. China; 5 Department of Forensic Medicine, Medical College of Soochow University, Suzhou, 215123 P.R. China

## Abstract

STRs vary not only in the length of the repeat units and the number of repeats but also in the region with which they conform to an incremental repeat pattern. Massively parallel sequencing (MPS) offers new possibilities in the analysis of STRs since they can simultaneously sequence multiple targets in a single reaction and capture potential internal sequence variations. Here, we sequenced 34 STRs applied in the forensic community of China with a custom-designed panel. MPS performance were evaluated from sequencing reads analysis, concordance study and sensitivity testing. High coverage sequencing data were obtained to determine the constitute ratios and heterozygous balance. No actual inconsistent genotypes were observed between capillary electrophoresis (CE) and MPS, demonstrating the reliability of the panel and the MPS technology. With the sequencing data from the 200 investigated individuals, 346 and 418 alleles were obtained via CE and MPS technologies at the 34 STRs, indicating MPS technology provides higher discrimination than CE detection. The whole study demonstrated that STR genotyping with the custom panel and MPS technology has the potential not only to reveal length and sequence variations but also to satisfy the demands of high throughput and high multiplexing with acceptable sensitivity.

## Introduction

Short tandem repeats (STRs) are the most widely used polymorphism markers in forensic community^1,2^. Polymerase chain reaction (PCR) and capillary electrophoresis (CE) are routine size-based methods for allele identification and follow simple conventions according to STR allele nomenclature^1–3^. The conventions are based only on the observed size variation generated by CE systems and do not account for sequence variations in the repeat motif and flanking sequences^3^.

Massively parallel sequencing (MPS), an interesting alternative to universal PCR-CE methods, may revolutionize the field of forensic STR genotyping^4^. Three commercial MPS assays (PowerSeq Auto system (Promega, Madison, WI, USA)^5,6^, ForenSeq™ DNA Signature Prep Kit (Illumina, San Diego, CA, USA)^7,8^, and Precision ID GlobalFiler NGS STR Kit (Thermo Fisher, Waltham, MA, USA)^9^), are now available for STR analysis in forensic community. These assays include 22, 27, and 29 forensic autosomal STRs, respectively. Studies^5–9^ demonstrated that MPS technology produces sequence data that provide a precise description of the repeat allele structure of STRs and variants that may reside within the amplified fragment or nearby flanking areas^4–9^, with multiple markers and multiple samples in one analysis.

Since Thermo Fisher Scientific provides AmpliSeq Designer and corresponding Ampliseq reagents for custom panel designing, we attempted to develop a panel that can sequence 34 autosomal STRs commonly applied in China’s forensic community with the Ion Torrent PGM platform and analyse the sequencing data following the newest recommendations issued by ISFG^4^, such as using the GRCh38 human reference genome instead of GRCh37, and defining the motif structure according to the NIST STRbase.

## Results and Discussion

In this study, the custom panel allows for simultaneously detection of up to 34 polymorphic forensic autosomal STRs and the sex determination locus of Amelogenin. Detail information of the 34 STRs and corresponding primers were attached as Supplementary Table S1. The Bed file and a Params file for analysis were accordingly programmed based on the coordinate positions and motif structures. We explored the MPS performance from sequencing reads analysis, concordance study and sensitivity testing, to document the performance capabilities and limitations of the custom panel.

### Sequencing reads analysis

9947A and 9948 (Promega, USA) were adopted as reference samples in this study. Libraries of them were pooled for two separate emPCRs and corresponding emPCR products which correspond to the library dilution point of 17% and 23% of template ISPs were sequenced on individual Ion 314 Chips. The sequencing data yielded concordant genotype results between each replicate and with the CE genotyping results. No significant differences in allele coverage (*p* = 0.0751) and allele coverage ratio (ACR) values (*p* = 0.1864) at the 34 STRs were observed between sequencing replicates, thus we combined the two batches of data together for analysis. The averaged depth of coverage (DoC) among the 34 STRs ranged from 1652 to 2760, with ACR values of heterozygotes ranging from 0.67 to 0.94. And Isoalleles, i.e., alleles of the same length but differing in sequence, were observed at D8S1179 of 9947A. Genotype of homozygote 13 was displayed with CE technology (Supplementary Fig. S1-A), while sequence heterozygote of [TCTA]_1_[TCTG]_1_[TCTA]_11_ and [TCTA]_13_ was recognized with MPS technology (Supplementary Fig. S1-B). And Sanger sequencing was conducted to verify the sequences (Supplementary Fig. S1-C).

**Figure 1:**
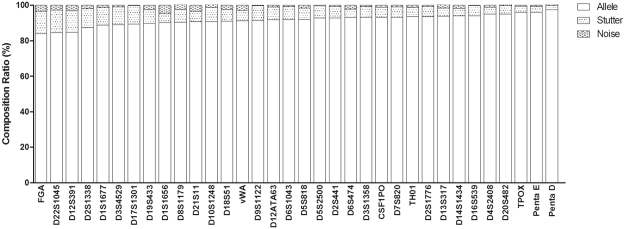
The allele/noise/stutter ratios at the 34 STRs with the double sequencing reads from the 200 individuals.

Sequencing reads observed at each locus can been divided into allele, stutter, and noise. Here, we analysed the allele/stutter/noise information of the 34 STRs with the double sequenced data from the 200 tested samples. Since a maximum of 25 samples can been sequenced on the Ion 318 v2 chip with this custom panel, the double tested 200 samples were sequenced on 16 separate runs. After filter out data from empty wells, polyclonal, tested fragments, adapter dimer and low quality signals, the usable reads of the 16 runs ranged from 68% to 87%. Although the run-to-run variation is unavoidable and do affect the constitution of sequencing reads, sequencing genotypes between each replicate of the 200 samples were concordant. Figure 1 shows the averaged composition ratios at the 34 STRs with the double-sequencing reads from the 200 individuals. The stutter ratios ranged from 2.425% (Penta D) to 12.686% (D22S1045), the noise ratios ranged from 0.097% (D17S1301) to 4.731% (D1S1656), while the allele ratios ranged from 83.888% (FGA) to 97.468% (Penta D). The averaged allele, stutter, and noise percentages were 91.64%, 6.808%, and 1.551%, respectively. Compared with sequencing data of corresponding STRs from the newest commercial MPS kits of Precision ID GlobalFiler^TM^ NGS STR Panel^9^ and Illumina® ForenSeq^TM^ DNA Signature Prep Kit^7,8^, significant differences were observed with the constitution ratios (data not shown). No noise signals was observed at TPOX, D6S1043, D2S1776, D3S1358, D16S539, and D7S820 with Precision ID GlobalFiler^TM^ NGS STR Panel^9^, while no noise signals was observed at Penta E with Illumina® ForenSeq^TM^ DNA Signature Prep Kit^8^. Since the evaluation of Precision ID GlobalFiler^TM^ NGS STR Panel was also performed on the Ion Torrent PGM platform, and same kits for emPCR and sequencing were used, the worse data of the custom panel presented here indicate that further optimization of the library primers should been explored in future studies.

**Figure 2:**
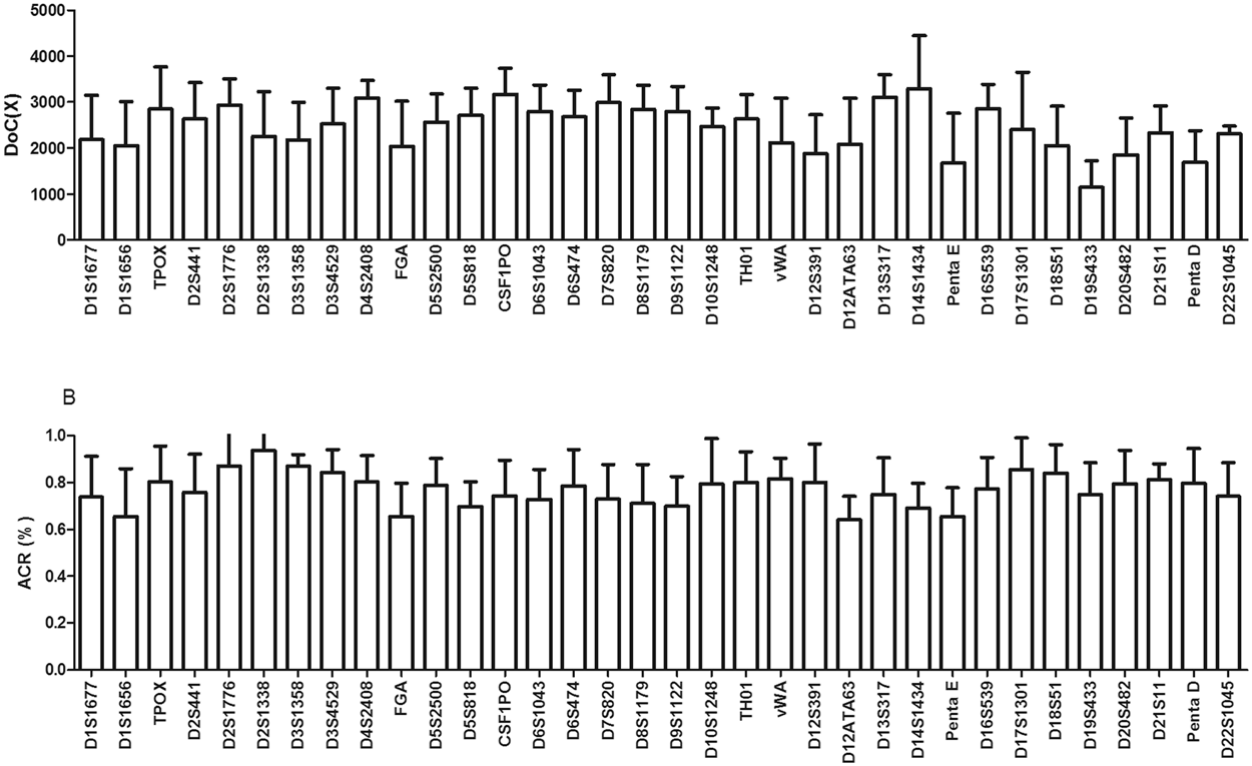
Sequencing performance of the 200 individuals. (**A**) Depth of coverage (Doc) of the 34 included STRs; (**B**) ACR values of the obtained heterozygous at the 34 STRs.

With the allele sequencing reads, we used the averaged values of Doc and ACR to evaluate the performance of the 34 STRs. Figure 2A illustrates the Doc information, while Fig. 2B shows the ACR values from the observed heterozygous balance at the 34 STRs. The mean DoC for the 34 loci ranged from a low value of 1144x ± 576.5 at D19S433 to a high value of 3284x ± 1163 at D14S1434. The mean ACR values ranged from 0.6418 ± 0.0998 (D12ATA63) to 0.9350 ± 0.0887 (D2S1338).

### Concordance study

A concordance study of the 200 DNA samples was first performed by comparing the genotypes from identical samples prepared and run in different sequencing reactions. No inconsistent genotype calling were observed between the double sequencing, although the Doc and ACR values of heterozygotes varied due to run-to-run variations. By Fisher’s exact test, no significant differences in ACR values (*p* = 0.1011) at the 34 STRs between each replicate sequencing were observed, indicating the variations of heterozygotes performance with different runs can be ignored.

A further concordance study was performed between CE genotypes and MPS data for all 200 individual samples and all 34 STRs, resulting in the evaluation of 6800 loci. In total, 346 and 418 alleles were obtained via CE and MPS technologies at the 34 STRs among the 200 individuals (Table 1). MPS technology did not identify additional alleles for 18 STR loci, with locus D21S11 showing the highest degree of variation. The additional 72 alleles were identified based on sequence differences in the same PCR fragment.

**Table 1:** Detected alleles and corresponding forensic parameters of the 34 STRs via MPS and CE detection (N = 200).

STR	MPS	PIC	PEtrios	PEduos	Allele Number	CE	PEtrios	PEduos
	Allele Number					PIC		
D1S1677	7	0.6108	0.4348	0.2464	7	0.6108	0.4348	0.2464
D1S1656	20	0.8676	0.7513	0.5895	16	0.8409	0.7070	0.5306
TPOX	7	0.6240	0.4031	0.2196	7	0.6240	0.4031	0.2196
D2S441	10	0.7691	0.5885	0.3892	7	0.7524	0.5772	0.3764
D2S1776	7	0.7102	0.5339	0.3329	7	0.7102	0.5339	0.3329
D2S1338	23	0.9224	0.8487	0.7305	12	0.8578	0.7356	0.5650
D3S1358	12	0.7334	0.5253	0.3260	8	0.6928	0.5133	0.3142
D3S4529	13	0.7810	0.6180	0.4225	8	0.7074	0.5306	0.3291
D4S2408	8	0.7230	0.5395	0.3373	8	0.7230	0.5395	0.3373
FGA	21	0.8622	0.7373	0.5687	21	0.8622	0.7373	0.5687
D5S2500 (AC008791)	10	0.7730	0.6048	0.4053	7	0.6708	0.4763	0.2793
D5S818	9	0.7480	0.5827	0.3823	9	0.7480	0.5827	0.3823
CSF1PO	12	0.7740	0.5997	0.4027	9	0.7177	0.5363	0.3359
D6S1043	17	0.8749	0.7620	0.6010	15	0.8674	0.7499	0.5838
D6S474	9	0.6950	0.5184	0.3194	9	0.6950	0.5184	0.3194
D7S820	9	0.6950	0.5953	0.3956	8	0.7545	0.5872	0.3868
D8S1179	17	0.8855	0.7815	0.6289	12	0.8427	0.7113	0.5320
D9S1122	11	0.7842	0.6175	0.4228	7	0.6641	0.4822	0.2864
D10S1248	9	0.7347	0.5592	0.3581	9	0.7347	0.5592	0.3581
TH01	7	0.6426	0.4299	0.2402	7	0.6426	0.4299	0.2402
vWA	13	0.8217	0.6726	0.4847	9	0.7950	0.6291	0.4319
D12S391	23	0.9234	0.8510	0.7342	12	0.8244	0.6854	0.5002
D12ATA63	10	0.7831	0.6119	0.4150	9	0.7366	0.5424	0.3428
D13S317	9	0.7901	0.6232	0.4251	9	0.7901	0.6232	0.4251
D14S1434	11	0.7437	0.5719	0.3721	8	0.6870	0.5068	0.3077
Penta E	10	0.8758	0.7649	0.6047	10	0.8758	0.7649	0.6047
D16S539	9	0.7766	0.6046	0.4050	9	0.7766	0.6046	0.4050
D17S1301	7	0.6702	0.4988	0.3000	7	0.6702	0.4988	0.3000
D18S51	17	0.8514	0.7303	0.5590	17	0.8514	0.7303	0.5590
D19S433	17	0.8180	0.6668	0.4796	17	0.8180	0.6668	0.4796
D20S482	6	0.6728	0.4963	0.2982	6	0.6728	0.4963	0.2982
D21S11	30	0.8949	0.7999	0.6575	22	0.8353	0.6942	0.5143
Penta D	12	0.7953	0.6433	0.4510	12	0.7953	0.6433	0.4510
D22S1045	6	0.7657	0.5931	0.3918	6	0.7657	0.5931	0.3918

Among the 200 samples, three samples were detect as homozygote “19” with Powerplex 21 (Promega, Wisconsin, USA) and Goldeneye^TM^ 20A (Goldeneye, co, Ltd, China) kits at D2S1338 locus; however, heterozygotes of 19/24, 17/19 and 19/24 were detected by MPS sequencing, respectively. In other words, allele drop-out may occurred at D2S1338 locus with the primers from the Powerplex 21 and Goldeneye^TM^ 20A kits by CE technology. Sanger sequencing were verified our conjecture (Supplementary Fig. S2). Since the primer information for the two commercial kits is confidential, we assume that the PCR primers may fail to amplify a particular allele due to variation in the STR flanking regions or primer binding site mutations of these samples.

We also found a sample genotyped with discordant homozygotes “11” and “11.1” at the CSF1PO locus with the Powerplex 21 kit (Promega, Wisconsin, USA) and Goldeneye^TM^ 20A kit (Goldeneye, co, Ltd, China), respectively. The MPS sequencing genotype of this sample was “11”. This phenomenon occurred due to insertion of a cytosine 128 bp downstream of the motif region. The results suggested that the reverse primer for the CSF1PO locus in the Goldeneye^TM^ 20A kit (Goldeneye, co, Ltd, China) should be moved to the region between the motif and the mutation site.

And a four-base deletion (rs561167308) in the 3′ flanking region of D13S317 was observed in two samples. Since the Indel was present outside the motif structure but within the amplified region, the PCR-CE detection gave alleles with one less repeat than MPS sequencing.

Above results reveal that no actual inconsistent results were existed among the 6800 genotypes. And STR sequencing information produced with MPS technology, include motif and flanking areas, can obtain a better understanding of STRs and reveal the flaws of current commercial STR kits.

### Sensitivity testing

**Figure 3:**
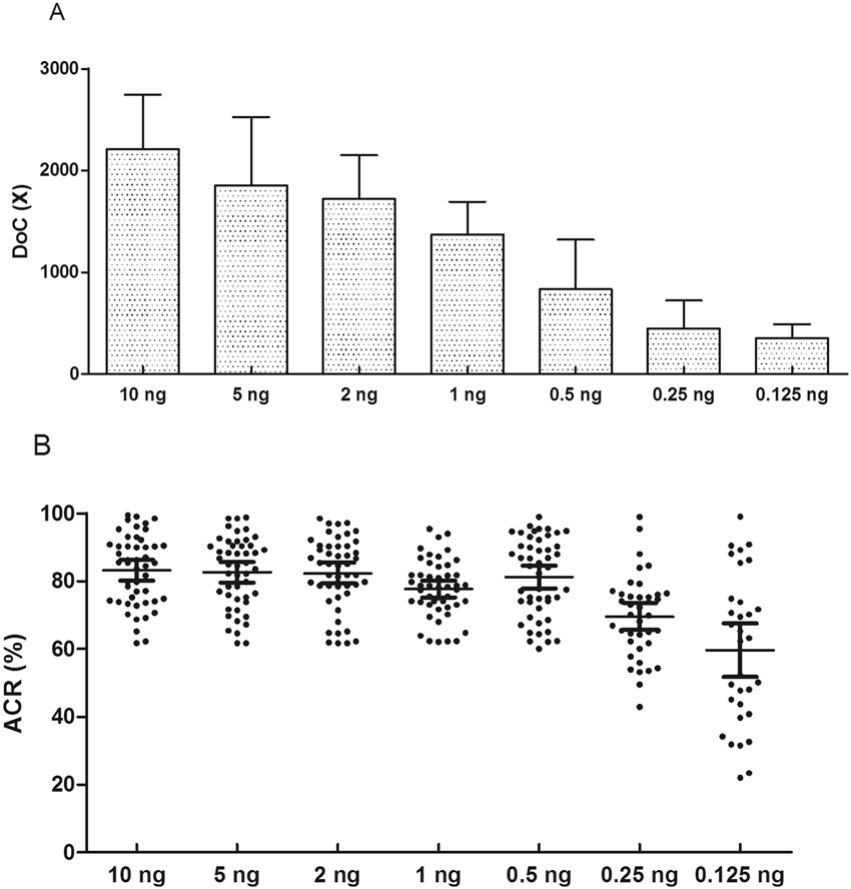
Sensitivity testing of series dilutions of control DNA 9948 from 10 ng to 0.125 ng. (**A**) Depth of coverage (Doc) of the seven dilutions; (**B**) averaged ACR values with 95% confidence interval for the 24 obtained heterozygous in the seven dilutions.

Sensitivity study can be defined as the ability to produce reliable profiles from a range of DNA quantities. Initial DNA of 10 ng was recommended for library preparation in the protocol ‘Ion AmpliSeq™ Library Preparation Revision A.0’ with our custom panel. To evaluate the sensitivity of this panel, libraries from a serial dilution (10 ng, 5 ng, 2 ng, 1 ng, 0.5 ng, 0.25 ng, and 0.125 ng) of control DNA 9948 were pooled in duplicate and sequenced on an Ion 316 v2 chip. The total obtained sequencing data was 373.28 MB. The CE genotyping results of 9948 at the 34 STRs were obtained by amplification of 0.5 ng DNA with Powerplex 21 (Promega, Wisconsin, USA), Goldeneye^TM^ 20A and Goldeneye^TM^ 22NC (Goldeneye, co, Ltd, China) kits individually. The 14 emPCR products were barcoded as 1–14. Concordant results were obtained between each replicate and with the CE genotyping results at the 34 STRs except when less than 0.5 ng of DNA was used. When 0.25 ng of DNA or less was used, allele drop-out was observed. Within the correct genotypes detected with 0.25 ng and 0.125 ng of DNA, heterozygous imbalance (ACR < 0.6) was observed. The mean DoC was 2208x ± 534 for the 10 ng library and 353x ± 139 for the 0.125 ng library (Fig. 3A). Figure 3B shows the performance of ACRs from the heterozygotes at the seven different concentrations (24 loci detected as heterozygotes). The variation of allelic balance of heterozygotes was greater in the experiments with lower amounts of DNA. The mean ACRs of heterozygotes were all greater than 60% when DNA ranged from 10 ng to 0.5 ng. When 0.25 ng and 0.125 ng of DNA were used, averaged ACR values of detected heterozygous are 69.58% and 59.71%, respectively. Above results demonstrated that the minimum DNA amount for this panel was 0.5 ng. The average DoC of the 34 STRs was 832x, while the average ACR value was 81.22% when 0.5 ng of DNA was used.

### Alleles and forensic parameters

STRs detected with MPS technology could provide sub-repeat variants that were undetected by PCR-CE typing. Among the 34 STRs, D21S11 was the most sequence-polymorphic locus, with 30 alleles ranging from 9 to 34.2 repeat units. In our study, isoalleles were detected at 16 STRs (Supplementary Table S2). The alleles and corresponding frequencies of the 418 sequenced alleles at the 34 STRs among the 200 individuals were listed in Supplementary Table S2. The forensic parameters obtained from both CE and MPS data were listed in Table 1. The statistical data were investigated regarding the increase in the number of effective alleles due to the presence of sequence variations in the STR repeat regions. The most significant changes in Power of Exclusion in trios (PEtrios) and Power of Exclusion in duos (PEduos) between MPS and CE were observed at D12S391, with +0.1657 and +0.234, respectively. As all 34 autosomal STR loci were independent from each other with linkage disequilibrium analysis, the combined forensic efficiency parameters were calculated based on the allelic frequencies. The Cumulative Power of Exclusion in duos (CPEduos) and in trios (CPEtrios) were 0.999 999 999 999 975 and 0.999 999 982 454 768 with CE methods, while the CPEduos and CPEtrios were 0.999 999 999 999 999 and 0.999 999 999 026 630 with MPS technology. Since the information obtained from MPS technology provided higher discrimination than those obtained from the CE detection, MPS method was expected to be particularly useful for parentage testing, by enabling the resolution of isoalleles, as well as distinguishing variants in flanking regions. However, as indicated by the ISFG guidelines, the current allelic frequencies obtained via MPS technology are not sufficient to quantify any new variations, thus comprehensive MPS databases is required to characterize the extent of the STR sequence variations for estimating the STR allele frequencies^4^.

## Methods

All involved biological samples were collected upon approval of the Ethics Committee at the Academy of Forensic Sciences, Ministry of Justice, P. R. China. A written informed consent was obtained for each participant. The main experiments were conducted at the Forensic Laboratory of Academy of Forensic Science, Ministry of Justice, P.R. China, which is an accredited laboratory by ISO 17025, in accordance with quality control measures. All the methods were carried out in accordance with the approved guidelines of Academy of Forensic Sciences, Ministry of Justice, P.R. China.

### STR selection and library-primer design

All STRs from Combined DNA Index System (CODIS), expanded CODIS, European Standard Set (ESS) and additional loci of ESS were listed as candidates. STRs from popular commercial kits of China’s forensic community (Powerplex 21 (Promega, Wisconsin, USA)^10^, Goldeneye^TM^ 20 A (Goldeneye, co, Ltd, China)^11^ and AGCU 21 + 1 (AGCU, co, Ltd, China)^12^) are also included. The start and stop coordinate positions (GRCh38 human reference genome) of motifs and structures referred to the newest forensic genetic nomenclature recommendations of ISFG^4^ and NIST STRbase^13^. AmpliSeq Designer was adopted for multi-primer designing, and the candidate targets were submitted to Thermo Fisher AmpliSeq primer design tool (http://www.ampliseq.com). The locus of SE33 was excluded from the final multi-primer designing process, remaining 34 STRs. Primers of the 34 targets were listed in Supplementary Table S1. The library length of them ranged from 243 to 310 base pairs (bps).

### Samples and library preparation

Blood samples were collected from 200 unrelated HAN individuals (100 females and 100 males) whose families had lived in Changzhou, China, for at least three generations. Each sample was extracted with the QIAsymphony SP DNA Extraction System (Qiagen, Germany) as recommended by the manufacturer^14^. Genomic DNA was quantified on an Applied Biosystems 7500 Real-time PCR System (Thermo Fisher Scientific, USA) with the Quantifiler Human DNA Quantification Kit (Thermo Fisher Scientific, USA). The concentrations of extracted DNA range from 15.28 to 49.31 ng/μL. All DNA samples were diluted to 10 ng/μL.

STR libraries were prepared with Ion AmpliSeq™ Library Kits 2.0 according to the Ion AmpliSeq™ Library Preparation (Revision A.0). The kit requires 10 ng of DNA per target amplification reaction. The library preparation system had a volume of 20 μL containing 4 μL of 5x Ion Ampliseq^TM^ HiFi Mix, 10 μL of primer pool, 5 μL of Nuclease-free water and 1 μL of above prepared DNA. Thermal cycling was performed on the GeneAmp 9700 System (Thermo Fisher Scientific, USA) with the Max ramping mode using the following conditions: (1) enzyme activation for 2 min at 99 °C and (2) 20 cycles of amplification at 99 °C for 15 s, 60 °C for 4 min, with a final hold at 10 °C. Two microliters of FuPa Reagent was used for partial digestion of the primer sequences. After adaptor ligation and library purification with the AMPure XP Reagent (Beckman Coulter, FL, USA), Ion Library Quantitation Kit (Thermo Fisher Scientific, USA) was used for accurate library quantification. Libraries were normalized to 10 pM and 8 μL pooled libraries was used for emulsion PCR (emPCR). This amount of emPCR product would correspond to the library dilution point among 15% to 30%, which ensure enough sequencing reads for data analysis. emPCR was performed using the pooled libraries on the Ion OneTouch^TM^ 2 instrument (Thermo Fisher Scientific, USA) with Ion PGM^TM^ Hi-Q^TM^ OT2 Kit (Thermo Fisher Scientific, USA), and the cycling setting was selected as “PGM: Ion PGM^TM^ Template OT2 400 Kit for Hi-Q^TM^.” Generated template-positive Ion Sphere Particles (ISPs) were enriched on the Ion OneTouch^TM^ ES instrument (Thermo Fisher Scientific, USA) according to the manufacturer’s recommendations.

### Ion Torrent PGM^TM^ sequencing

Samples sequenced on the same chip were pooled in equimolar ratios prior to sequencing. Considering the chip content and coverage depth, we sequence a maximum of 25, 14, and 3 samples in parallel on the Ion 318 v2, 316 v2 and 314 v2 chips. Each sample was sequenced twice in this study. Sequencing was performed on the Ion Torrent PGM^TM^ platform (Thermo Fisher Scientific, USA) using the Ion Torrent PGM^TM^ Hi-Q^TM^ Sequencing Kit (Thermo Fisher Scientific, USA); the number of flows was “850”, and the nucleotide flow order was “Samba Gafieira,” which improved the end-to-end success rates and signal-to-noise ratios for STR sequencing.

In this study, 200 individual samples were involved for sequencing performance evaluation of the custom panel. For these samples, we pooled 25 different DNA samples for each emulsion PCR (emPCR) and each sequencing reaction; thus, a total of 16 Ion 318 v2 chips were used. For sensitivity testing, purchased 9948 of 10 ng/µL (Promega, USA) was serially diluted to generate DNA concentrations of 5, 2, 1, 0.5, 0.25 and 0.125 ng/µL, and 1 µl of each concentration was added to the library preparation system. Thus, the DNA input for sensitivity testing ranged from 10 ng to 0.125 ng. The samples were subject to 25 target amplification cycles for <1 ng of DNA input. Above DNA were prepared by two independent operators in parallel, thus 14 libraries were generated. To avoid variations in emPCR and sequencing runs, libraries were pooled for one emPCR and labeled by different barcodes to conduct template preparation and then sequenced on one Ion 316 v2 chip.

### Data processing

Raw data were processed with Ion Torrent Suite Software v4.4.0 (Thermo Fisher Scientific, USA), and STR sequence calling was handled with the HID STR Genotyper v4.0 plugin equipped with a self-programmed BED file and a Param file. The analytical threshold of 250 sequencing reads was applied. After the analysis, a PDF report with detailed genotypes, coverage, sequences and coverage plots for each sample at each STR locus and an Excel file listing the barcode, sample, locus, allele, status, coverage and sequence information were obtained.

The MPS-STR reads observed at each locus were divided into allele, stutter and noise reads. Stutters were defined as sequences in which one or two motifs were shorter or longer than the parent allele. Noise was defined as reads that were not alleles or stutters, i.e., PCR/sequence errors. Allele/stutter/noise percentages were determined by dividing the number of reads containing the allele/stutter/noise by the total number of reads for each locus. DoC and ACR parameters were used to evaluate the STR sequencing performance. The ACR parameter was determined by dividing the lower-coverage allele by the higher-coverage allele at heterozygous genotypes.

Sequence allelic frequencies were assessed with direct counting methods. Statistical parameters of polymorphism information content (PIC), exclusion power in duos (PEduos) and exclusion power in trios (PEtrios) to evaluate the forensic efficiency were calculated using the formulas listed in references^15,16^.

### CE genotyping

Control samples (9947 A and 9948) and the 200 blood samples were amplified with the Powerplex 21 (Promega, Wisconsin, USA), Goldeneye^TM^ 20 A and Goldeneye^TM^ 22NC (Goldeneye, co, Ltd, China) kits according to the manufacturers’ guidelines. The amplified products were separated and detected on an Applied Biosystems 3130*xl* Genetic Analyzer (Thermo Fisher Scientific, USA). Raw data were analyzed using GeneMapper ID Software v3.2.1 (Thermo Fisher Scientific, USA). The analytical threshold used for CE analysis was set to 200 relative fluorescence units (RFU). The alleles determined with PCR-CE method were compared with the allele calls from the sequencing data. And the inconsistent results were verified by Sanger sequencing.

## Electronic supplementary material

Supplementary Tables S1-S2 and Figure S1-S2

Supplementary Table S1

Supplementary Table S2
