# Supplementary material for: Sequence investigation of 34 forensic autosomal STRs with massively parallel sequencing
Source: Sci Rep. 2018 May 1;8:6810. doi: 10.1038/s41598-018-24495-9 (PMC5931506; doi:10.1038/s41598-018-24495-9)
Supplement: Supplementary file 1 — Supplementary Tables S1-S2 and Figure S1-S2 [file 41598_2018_24495_MOESM1_ESM.pdf]

Supplementary Tables S1-S2 and Figure S1-S2

Sequence investigation of 34 forensic autosomal STRs with massively parallel sequencing

Suhua Zhang[1], Yong Niu[2], Yingnan Bian[1], Rixia Dong[3], Xiling Liu[1], Yun Bao[1], Chao Jin[4], Hancheng Zheng[5], Chengtao Li[1]*

[1] Shanghai Key Laboratory of Forensic Medicine, Shanghai Forensic Service Platform, Institute of Forensic Sciences, Ministry of Justice, P.R. China, Shanghai 200063, P.R. China

[2] Criminal Investigation Department, Ministry of Public Security, P.R. China, Beijing 100741, P.R. China

[3] The Affiliated Guangji Hospital of Soochow University, Suzhou 215008, P.R. China

[4] Shanghai OE Biotechnology Co, Ltd, Shanghai 201114, P.R. China

[5] Department of Forensic Medicine, Medical College of Soochow University, Suzhou 215123, P.R. China

*Corresponding author.

E-mail: lichengtaohla@163.com;

ORCID: 0000-0001-6852-9144.

Legends:

Supplementary Table S1 Detail information of 34 autosomal STRs and corresponding primers

Supplementary Table S2 Observed alleles and corresponding motif sequences from the 200 individuals sequenced with the custom MPS-STR panel. Bold nucleotides are not included in the repeat number designation.

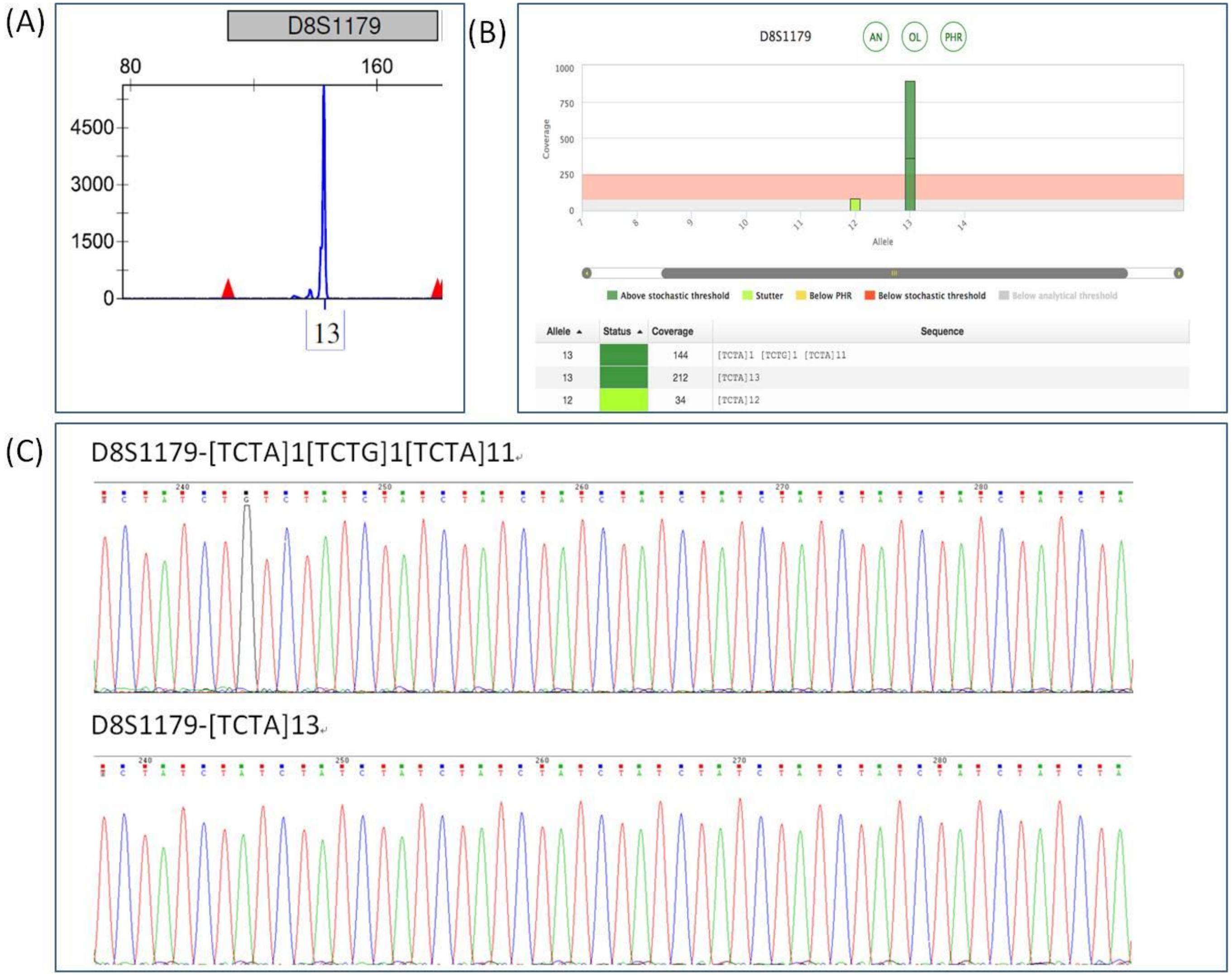
(A)
D8S1179
80
160
4500
3000
1500
0
13
(B)
D8S1179
AN
OL
PHR
1000
750
500
250
0
Coverage
7
8
9
10
11
12
13
14
Allele
Above stochastic threshold
Stutter
Below PHR
Below stochastic threshold
Below analytical threshold
Allele Status Coverage Sequence
13 144 [TCTA]1 [TCTG]1 [TCTA]11
13 212 [TCTA]13
12 34 [TCTA]12
(C)
D8S1179-[TCTA]1[TCTG]1[TCTA]11
240
250
260
270
280
D8S1179-[TCTA]13
240
250
260
270
280

Supplementary Fig S1 Genotype of D8S1179 of control DNA 9947A (A) Genotypes of 13 detected by CE; (B) Isoalleles detected by MPS technology with the custom panel; (C) Sanger sequences of [TCTA]1[TCTG]1[TCTA]11 and [TCTA]13 was recognized.

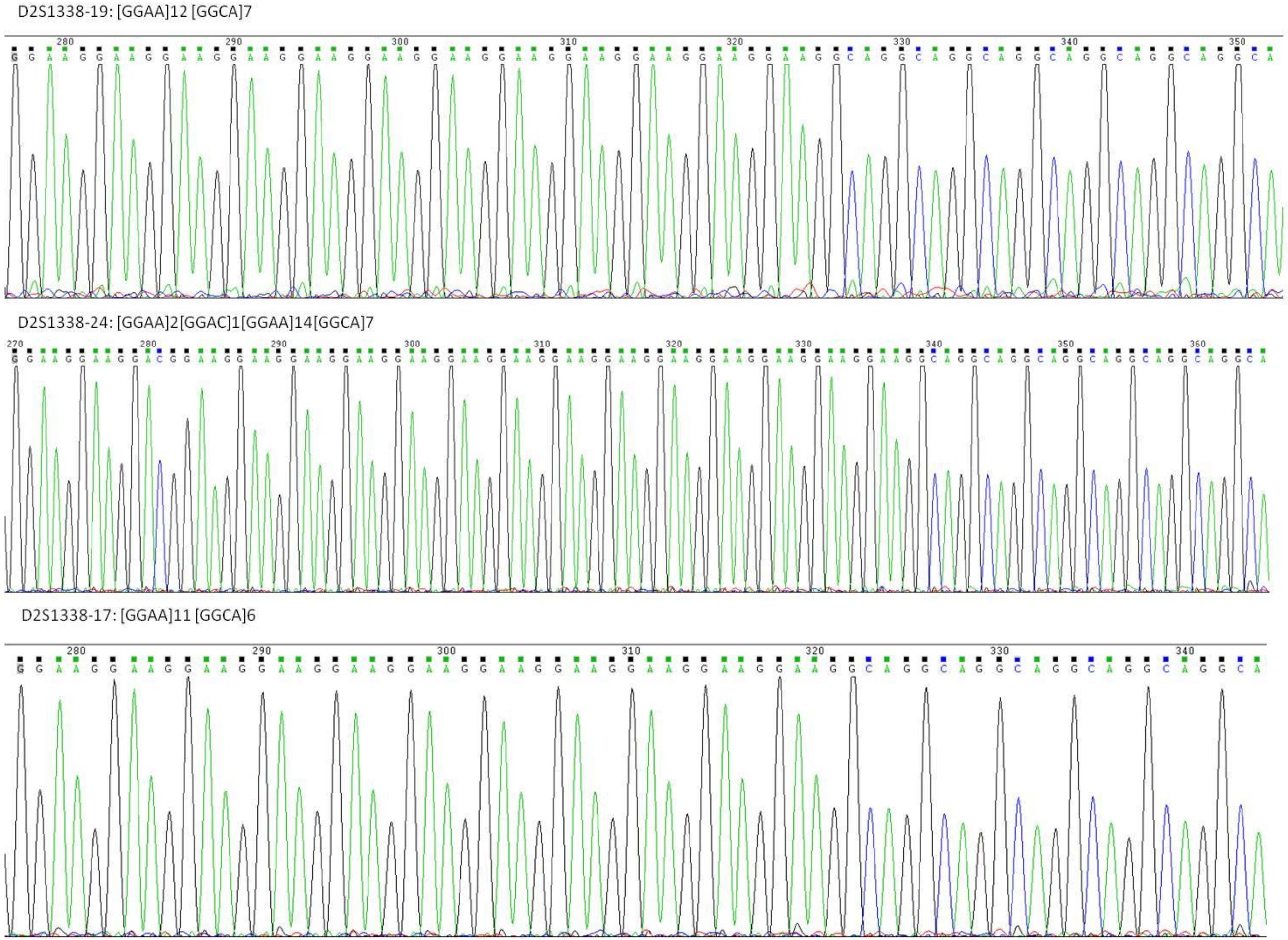

Supplementary Fig S2 Sanger sequencing results of three homozygotes of "19" detected by CE. Two new allele sequences of 24 and 17 were observed.
